# T7 Phage as an Emerging Nanobiomaterial with Genetically Tunable Target Specificity

**DOI:** 10.1002/advs.202103645

**Published:** 2021-12-16

**Authors:** Hui Yue, Yan Li, Mingying Yang, Chuanbin Mao

**Affiliations:** ^1^ School of Materials Science and Engineering Zhejiang University Hangzhou Zhejiang 310027 P. R. China; ^2^ Institute of Applied Bioresource Research College of Animal Science Zhejiang University Yuhangtang Road 866 Hangzhou Zhejiang 310058 P. R. China; ^3^ Department of Chemistry and Biochemistry Stephenson Life Science Research Center Institute for Biomedical Engineering, Science and Technology University of Oklahoma 101 Stephenson Parkway Norman Oklahoma 73019‐5251 USA

**Keywords:** genetic engineering, precision medicine, T7 phage, targeted theranostics

## Abstract

Bacteriophages, also known as phages, are specific antagonists against bacteria. T7 phage has drawn massive attention in precision medicine owing to its distinctive advantages, such as short replication cycle, ease in displaying peptides and proteins, high stability and cloning efficiency, facile manipulation, and convenient storage. By introducing foreign gene into phage DNA, T7 phage can present foreign peptides or proteins site‐specifically on its capsid, enabling it to become a nanoparticle that can be genetically engineered to screen and display a peptide or protein capable of recognizing a specific target with high affinity. This review critically introduces the biomedical use of T7 phage, ranging from the detection of serological biomarkers and bacterial pathogens, recognition of cells or tissues with high affinity, design of gene vectors or vaccines, to targeted therapy of different challenging diseases (e.g., bacterial infection, cancer, neurodegenerative disease, inflammatory disease, and foot–mouth disease). It also discusses perspectives and challenges in exploring T7 phage, including the understanding of its interactions with human body, assembly into scaffolds for tissue regeneration, integration with genome editing, and theranostic use in clinics. As a genetically modifiable biological nanoparticle, T7 phage holds promise as biomedical imaging probes, therapeutic agents, drug and gene carriers, and detection tools.

## Graphical abstract

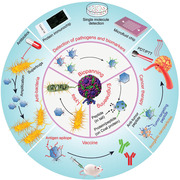

## 1 Introduction

Viruses are biological nanoparticles in nature. Numerous viruses exist on the Earth with an estimated number of ≈10^31^.^[^ ^1^ ^]^ Quite a few viruses play an irreplaceable role in Global Biogeochemical Cycles and can only replicate inside the living cells.^[^ ^2^ ^]^ Various living organisms, ranging from flora, fauna, to microbes (such as archaea and bacteria), could be infected by viruses.^[^ ^3^ ^]^ Among all of the viral population, bacteriophages, or phages for short, infect and replicate in fungi, algae, bacteria, actinomyces, spirochetes, and archaea. Bacteriophages, first identified in staphylococcus and shigella bacteria in 1915,^[^ ^4^ ^]^ are natural antibacterial agents, making this group of viruses unique.^[^ ^5^ ^]^

There are two major types of phages, lytic and temperate. Lytic phages generally consist of a front end and a flexible tail, whereas most temperate phages have long filamentous structures. Unlike temperate phages, the lytic phages kill bacteria when infecting bacteria. The lytic phages are mainly named after the lysis ability of host cells during the infectious cycle. Typical lytic phages include T1–T7 phages, named after the order of their discovery, are divided into T‐even (2, 4, 6) series and T‐odd (1, 3, 5, 7) series due to their different chemical compositions.^[^ ^6^ ^]^ Among them, T7 is a kind of typical lytic phage in the family of podoviridae, which is characterized by noncontractile and short tails.^[^ ^7^ ^]^ It is an ideal phage for experimentation due to the short replication cycle, efficient cytoplasmic protein assembly, high cloning efficiency, easy operation, and convenient storage.^[^ ^8^ ^]^

Phages are assembled from proteins (forming outer capsid) and the protein‐encoding nucleic acids (protected inside the capsid). Foreign genes, encoding peptides or proteins, can be inserted into genes of the appropriate genome positions of the phages through genetic engineering, allowing the foreign peptides or proteins to be fused to the capsid proteins of the phage. This principle further enables the generation of high‐quality libraries made of phage clones, with each displaying a unique peptide or antibody fragment.^[^ ^9^, ^10^ ^]^ Constructing this system is facile and not labor‐intensive.^[^ ^11^ ^]^ There exist various phage display systems, such as M13, T4, and T7 systems. M13 phage is a typical temperate phage that could be pictured as a filamentous nanofiber composed of five structural capsid proteins. T4 and T7 phages are the two most studied lytic phages composed of an icosahedral head and flexible tails instead of a long filamentous structure. The structural difference between T4 and T7 phage is that T7 phage has a short tail while T4 phage has a long tail, a base plate and long tail fibers. The most commonly used vector is the M13 phage. However, the T7 system has several advantages over the M13 system.

The main differences between T7 and M13 bacteriophages lie in the structures and ways in which mature viruses are released from host cells. First, the T7 phage is directly released from the host cells after its assembly in the cytoplasm and disruption of the host cells, enabling it to display a large polypeptide or even a protein. However, M13 phage is assembled in the periplasm and must be secreted by the cell membrane without disrupting the host cells. Thus, displaying larger polypeptides or proteins on the M13 phage is challenging and sometimes even impossible. Second, the recombinant T7 phage has great stability, even with a foreign gene more than 1 kb inserted;^[^ ^12^ ^]^ while the amplification of M13 phage is much more limited by the inserted gene length, which is less than 1500 bp, and the stability of the genome would be reduced after foreign DNA is inserted.^[^ ^13^ ^]^ The different DNA structures between T7 and M13 phages cause different performances. The T7 phage is composed of double‐stranded DNA (ds DNA), while M13 phage consists of single‐stranded DNA (ssDNA). The dsDNA has good stability and is not prone to mutation during replication.^[^ ^14^ ^]^ Besides, T7 phages show great stability under harsh ambient conditions (e.g., high acid concentration or temperature), which benefits efficient and high throughput biopanning, a method to obtain phages with target‐binding affinity.^[^ ^15^, ^16^ ^]^ Third, T7 phage grows fast and generates plaques within 3 h, saving much time for cloning and screening, in contrast to the slow growth and plaque forming (12 h needed) of M13 phage.^[^ ^17^ ^]^ Especially, the highly adaptable T7 phage can be isolated within a latent period of only 11 min under the condition of high yield medium and constant temperature of 37 °C. The number of the adaptive phage effectively is increased by 10^13^ after growth for an hour.^[^ ^18^, ^19^ ^]^ Fourthly, the lysis of bacteria by T7 phage does not rely on the secretory of protein. In addition, a foreign cDNA library can be inserted into the genome of T7 phages directly, which would express as capsid proteins.^[^ ^20^ ^]^

**Figure 1:**
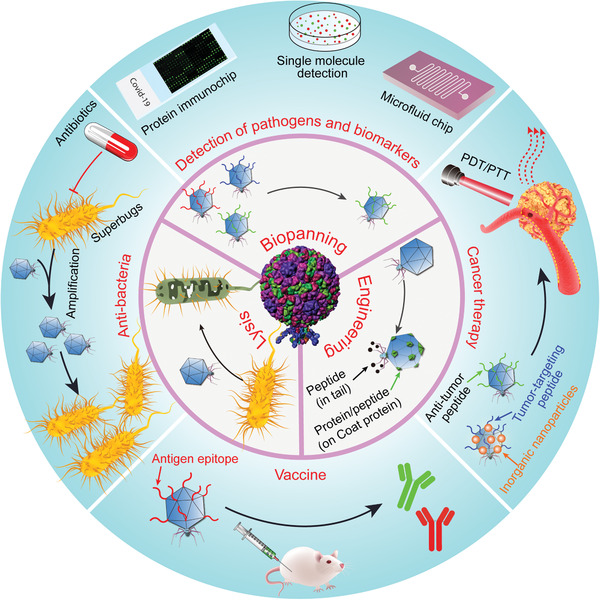
Overview of T7 phage‐based nanomedicine strategies for the detection of serological biomarkers and bacterial pathogens, antibacteria therapy, targeted cancer therapy, and effective vaccinations. T7 phage can be genetically engineered to display targeting motifs that recognize cells or tissues with high affinity, enabling it to serve as a disease site‐homing drug, a gene delivery carrier, a vaccine, an antibacterial agent, or a probe for detecting biomarkers and pathogens.

Due to these benefits of T7 phage, scientists have employed it as a biological nanostructure to develop its theranostic applications, forming the basis of this review. As shown in **Figure** **1** and **Tables** **1** and **2**, there exist several sophisticated strategies for T7 phage‐based nanomedicine. First, the T7 phage display system could be utilized to find out disease‐targeting biomarkers. The affinity peptide could be obtained through in vivo and in vitro biopanning. Second, identification of the cell‐ or tissue‐specific homing motifs can be achieved by in vivo biopanning of the T7 phage, which has been proved a potent method. After the injection of phage libraries intravenously into mice, T7 phage is circulated. Some phage particles binding to specific organs or tissues are collected. Third, affinity agents, especially phage particles, can be presented on the surface of nanoparticles to obtain the target‐specific nanoparticles. Fourth, T7 phage is also considered a good candidate for introducing genes into eukaryotic cells since it can tolerate the insertion of long foreign gene sequences into their genome, facilitating the development of new gene therapy and vaccines. Fifth, T7 phage can improve targeted drug delivery. Drugs can either be bound to its surface or packaged within it. Last but not least, genetically engineered T7 phage can be employed for targeted antibacterial therapy. Modifying the genes of T7 phage can inactivate the bacteria‐resistant genes.^[^ ^20^, ^21^, ^22^ ^]^ So far, there has not been a review dedicated to the theranostic applications of T7 phage.

**Table 1:** Outline of disease/pathogen detection based on T7 phage

Application	Diseases/pathogens	Notes	Ref.
Serological biomarkers	Cystic fibrosis	With detection sensitivity of 0.999 and specificity of 0.959	[23]
	Mycobacterium tuberculosis infection	With detection sensitivity of 1 and specificity of 1	[24]
	Foot‐and‐mouth disease	Identifying functional epitope VP1159‐170	[25]
	Alzheimer's disease (AD)	Identifying a panel of AD autoantibodies	[26]
	Autoimmune retinopathy	By immunoprecipitation sequencing (PhIP‐Seq)	[27]
	Lung tumor	Constructing lung tumor protein chip based on T7 phages	[28]
	Melanoma	By T7 phage display antigen microarrays	[29]
Detection of E. coli	In water	Detection of less than 10 CFU mL^−1^ *E. coli* in 3 h	[30]
	In drinking water	Detection of 1 × 10^4^ CFU mL^−1^ within 2.5 h	[31]
	In beverages	Detecting alkaline phosphatase	[32]
	In food and water	Detecting alkaline phosphatase maltose‐binding protein	[33]
	In food matrices	Detecting the overexpressed enzymes	[34]
	*E. coli*	Detecting the overexpressed luciferase	[35]
	*E. coli*	For phages and for antibiotic resistance testing	[36]
Detection of pathogens	Viable bacterial pathogens	By T7 phages carried tobacco etch virus (TEV) protease	[37]

**Table 2:** Review of targeted therapy based on T7 phage

Application	Targets	Notes	Ref.
In utero tissue‐targeted therapies	Fetal tissues	T7 phages crossing the placental barrier and reaching fetal tissue	[38]
Inhibitory peptide	Cytidine triphosphate synthase 1 (CTPS1)	CTpep‐3 binding to the amidoligase (ALase) domain on CTPS1	[39]
	Lethal factor (LF)	Pepsin A3 pre protein (PAP) affinity to both types of LF	[40]
	Phospholipid hydroperoxidase glutathione peroxidase (GPX4)	One peptide binding to near Sec73 of catalytic site and two peptides binding to another site on GPX4	[41]
	K‐Ras	Krpep‐2d inhibiting signal of K‐Ras and proliferation of cancer cell A427	[42]
Antibacteria	Pseudomonas aeruginosa and *E. coli*	T7 phages displayed AiiA lactonase degrading AHLs	[43]
	Staphylococcus aureus	The scFv antibody against *S. aureus* from the spleen phage display library	[44]
	Bacteria in fresh produce	Whey protein isolate (WPI) coating providing antimicrobial activity	[45]
Vaccine	Avian influenza virus	T7 phages displayed M2e peptides of avian influenza A virus	[46]
	Lewis lung carcinoma	T7 phages expressing xenogenic VEGF on the capsid	[47]
	Foot‐and‐mouth disease	T7 phages expressing a fused G‐H loop peptide (T7‐GH)	[48]
	Vero cells	T7 phages expressing Tat peptide and carried eukaryotic expression box	[21]
Cancer therapy	MCF‐7 cancer cells	T7 phages displayed 6His‐spacer‐RGD4C peptide and loaded by copper	[49]
	PC3 cells	T7 phage bound with both PC3 cell surface and Au binding peptide	[50]
	Roxithromycin (RXM)	Roxithromycin (RXM) binding to extracellular domain on angiomotin (Amot)	[51]
Disease therapy	Tuberculosis	Gp2 binding to mycobacterial RNAP and inhibiting transcription	[52]
	Central nervous system diseases	Fc‐fused TrkB (TrkB‐Fc) to activate homodimerize TrkB	[53]

## 2 Structure Biology of T7 Phage as a Nanobiomaterial

**Figure 2:**
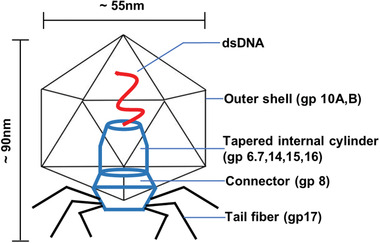
Biological structures of T7 bacteriophage. The T7 bacteriophage contains an icosahedral capsid, a noncontractible tail, and six fibers attached to it. The main functional parts of the outer shell (capsid) include gp10A and gp10B, and protect the linear dsDNA inside the phage. The tapered internal cylinder helps the DNA injected into host cells. The six tail fibers can assist the T7 phage to anchor onto the host cell surface to begin the bacterial infection. The connector with the ring structure is made of a few copies of gp8, forming a connection between the tail and head.

**Figure 3:**
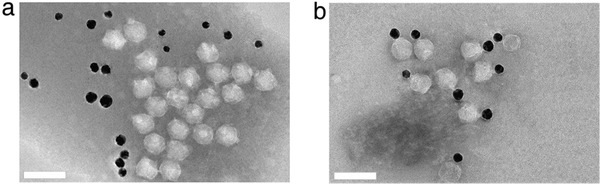
Transmission electron microscopy (TEM) of two kinds of T7‐gold nanoparticles. a) TEM image showing there exist no binding affinity between T7 phages without gold‐binding peptide displayed and gold nanoparticle. b) TEM image showing there exist binding affinity between T7 phages with gold‐binding peptide displayed and gold nanoparticle. Scale bar, 100 nm. Reproduced with permission.^[^ ^62^ ^]^ Copyright 2015, Springer Nature.

**Figure 4:**
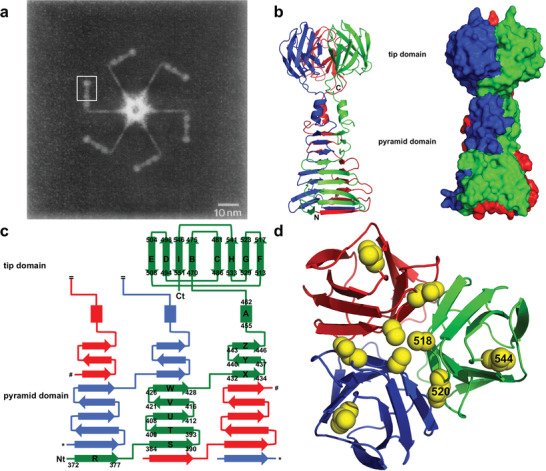
Crystal structure of T7 phage gp17 by electron microscopy. a) The electron microscopy image of the tail with high resolution. The box region was one‐sixth part of the tail's symmetrically equivalent regions. Reproduced with permission.^[^ ^72^ ^]^ Copyright 1988, Elsevier. b) Space‐filled representation (right) and Ribbon diagram (left) of gp17. The colors of the three parts were blue, green and red, respectively. c) The topology. The a‐helices and ß‐strands were displayed as rectangles and arrows, respectively. The beginning and end residues of secondary structural elements are labeled as green monomers. d) Vertical view of the tip domain. Reproduced with permission.^[^ ^56^ ^]^ Copyright 2012, National Academy of Sciences.

Both high precision electron microscopy and crystallography have been used to reveal the structure of phage particles and their proteins.^[^ ^54^, ^55^, ^56^, ^57^, ^58^, ^59^ ^]^ The detailed structure of T7 phage is represented in **Figure** **2**. The T7 phage has perfect structural symmetry, with dsDNA encapsulated in the capsid structure.^[^ ^60^, ^61^ ^]^ The inner diameter of its icosahedral head is 55 nm; the length and diameter of the tails attached to the head are 28.5 and 19 nm, respectively.^[^ ^60^ ^]^ A direct comparison between T7 phage and gold nanoparticles clearly shows that the T7 phage is indeed a nanoparticle and can bind a gold nanoparticle when engineered to display a gold binding peptide in **Figure** **3**.^[^ ^62^ ^]^ The head of T7 phage consists of a linear dsDNA (39936 bp) and 6 capsid proteins, including gp10A, gp10B, gp8, gp11, gp12, and gp17. A total of 415 proteins constitute each head.^[^ ^15^ ^]^ Gp10A and gp10B are mainly connected with fusion capsid protein. The cylindrical structure in the capsid is composed of gp14, gp15, and gp16,^[^ ^63^ ^]^ and linked to a connector (gp8 of circular dodecamer).^[^ ^14^ ^]^ The tail of T7 phage mainly consists of a large protein gp12 with six copies and a small protein gp11.^[^ ^55^ ^]^ The end of the tail is connected with six fibers; each fiber is composed of gp17 protein with three copies.^[^ ^56^ ^]^ T7 phage recognizes the host by its tail and tail fibrin, called receptor binding protein (RBPs).^[^ ^64^ ^]^ **Figure** **4** shows the crystal structure of gp17 determined by electron microscopy. Tail fibers of T7 phages are assembled from elongated homologous trimers and take on the responsibility for identifying the host cell.^[^ ^57^ ^]^ Presumably, tail‐tube proteins may mediate a second, irreversible, bacterial membrane interaction decision. DNA transfer from phage to the host is mediated by elongation of gp14–16.^[^ ^58^ ^]^ Other components, including gp6.7, gp7.3, and gp13, are necessary for infection, although their locations have not been determined.^[^ ^65^, ^66^ ^]^

At 65 °C, the loss of the capsid tail and the release of genomic DNA are revealed by atomic force microscopy (AFM) based nanomechanical measurements; thus, the T7 is unstable at this temperature. However, further heating to 80 °C results in better mechanical stability, possibly due to partial denaturation of the cap protein. Importantly, although the DNA loss caused by heat treatment destroys the stability of T7 phages, their capsid can withstand higher temperatures with their intact structures. Therefore, partial denaturation within the overall structure of the virus capsid may lead to a stabilization effect.^[^ ^67^ ^]^

When a T7 bacteriophage infects cells, some capsid proteins inside the phage would be ejected toward the surface of host cells first. Subsequently, they would be assembled into an ejector that helps the phage DNA enter the host cells.^[^ ^68^ ^]^ The phage DNA is then subjected to transcription and translation to form proteins in bacteria.^[^ ^68^ ^]^ The structural proteins are assembled with the progeny DNA to form T7 phages. At last, lysozyme is produced during the assembly process and used to lyse the host cells to release the mature viruses.^[^ ^69^ ^]^ The process described above goes very fast in practice, which even takes only a few minutes to complete, and its speed depends mainly on the adaptability of phages.^[^ ^70^ ^]^ For example, the life cycle of T7 phages is 17 min at 37 °C, which is relatively short. The life cycle means the total time from infecting the host cell to releasing new phages.^[^ ^71^ ^]^ All these special abilities have allowed the T7 phage to hold promise in antibacterial applications besides many others.

## 3 Genetic Engineering of T7 Phage

### 3.1 Genetic Engineering to Display Peptides and Proteins

**Figure 5:**
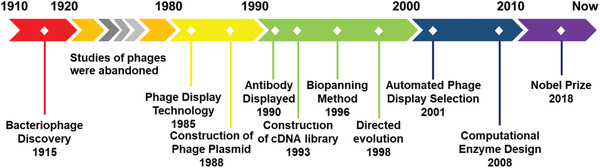
The development history of the phage display. Ten milestone processes for the display system are indicated along the timeline.

Bacteriophage display technology introduces the DNA sequence of exogenous polypeptides into the appropriate genes of phage DNA, making the foreign proteins displayed on the capsid along with the bacteriophage reassembly.^[^ ^15^, ^73^, ^74^, ^75^, ^76^, ^77^ ^]^ With the reassembly of progeny phages, relative spatial structure, and biological activity are kept in these phages. In 1985, bacteriophage display technology was first invented by George Smith.^[^ ^78^, ^79^ ^]^ Over the past 30 years, the technology has undergone several essential developments (**Figure** **5**), with significant advances in different target types and phage display methods.^[^ ^79^, ^80^, ^81^, ^82^, ^83^, ^84^, ^85^, ^86^, ^87^, ^88^, ^89^ ^]^

Two kinds of genetic systems have been formed to display polypeptides and proteins. The first one involves inserting foreign genes into the capsid protein genes of phage.^[^ ^90^ ^]^ The second system is more commonly used now. In this method, proteins are expressed using a single plasmid (also known as a phagemid), which is isolated from phage replication. The plasmid includes genes encoding foreign proteins fused to a capsid protein, while other protein‐coding genes are not included.^[^ ^91^, ^92^ ^]^ In addition, Liu et al. have developed ΦC31 integrase with enhanced activity and specificity of foreign gene knock‐in,^[^ ^93^ ^]^ representing a new way for integrating foreign DNA into the T7 phage genome.

**Figure 6:**
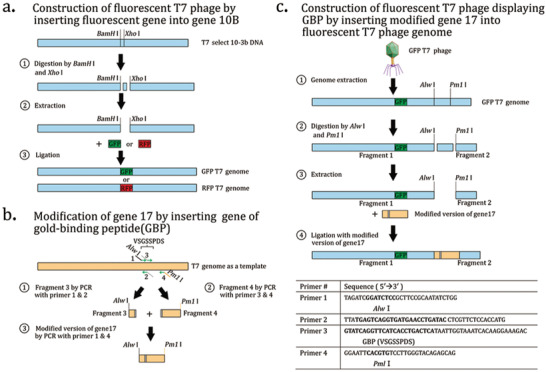
The schematic of establishing fluorescent T7 phage with or without a peptide (GBP) displayed. a) The procedure for generating fluorescent T7 phage by inserting the GFP/RFP gene into the gene 10B of the T7 phage. b) The steps to obtain the modified version of gene 17 with GBP incorporated. c) Formation of fluorescent T7 phage displaying GBP by inserting modified gene 17 into fluorescent T7 phage genome. Reproduced with permission.^[^ ^62^ ^]^ Copyright 2015, Springer Nature.

T7 bacteriophage is a kind of DNA virus with a cleavage life cycle. Since T7 phage is assembled in the cytoplasm and released by bacteria lysis, it is independent of the secretory mechanism of *Escherichia coli* (*E. coli*).^[^ ^94^ ^]^ Gp10A and gp10B within the head of T7 phage have been proved useful in the T7 bacteriophage display technology because foreign polypeptides can be fused to 10A (344 amino acid residues) or 10B (397 amino acid residues). Besides, the 10B region exists on the phage surface, so the technology is usually to insert foreign genes into the C‐terminal P10B.^[^ ^55^ ^]^ Thus, T7 phages could display a short peptide (e.g., 50‐mer) in high copy or a protein (e.g., 1200 amino acid residues in length) in low copy (0.1–1/phage) or medium copy (5–15/phage). Therefore, the T7 bacteriophage display system is widely applied to screen affinity peptides and proteins with different molecule weights.^[^ ^95^ ^]^ In addition to the gp10 region, foreign gene sequences can also be inserted into other regions such as gp17.^[^ ^62^ ^]^ **Figure** **6** represents the schematic diagram of the construction of recombination T7 phage by inserting a fluorescent gene into gene 10B and inserting gene of gold‐binding protein into gene 17.^[^ ^62^ ^]^ First, the vector (T7 select 10‐3b) was digested using *BamH*I/*Xho*I and ligated with GFP/RFP gene to form the T7‐GFP/T7‐RFP gene, respectively. Then the over‐lap PCR was used to insert the GBP‐binding peptide into gene 17 of T7 phage. After that, the T7‐GFP/T7‐RFP gene and the GBP‐binding peptide‐inserted gene17 were digested with *Alw*I/ *Pwl*I, and ligated together to form the double‐display T7 phage DNA.

In short, T7 phage display system has unique advantages over other display systems. First, large‐size foreign proteins containing 1000 amino acids could be incorporated into capsids.^[^ ^71^ ^]^ Second, T7 phage keeps excellent stability under different harsh conditions, such as high solution concentration and temperature, even in the case of denaturing agents. What is more, it grows very fast and thus can be mass‐produced in a short time. Moreover, the T7 phage library can bear more significant polypeptide diversities than the M13 phage library.^[^ ^95^ ^]^

### 3.2 Generation of Peptide‐Displaying Libraries

The capability of bacteriophage display technology mostly depends on the phenotype‐to‐genotype linkage between the interested polypeptides or proteins displayed on the capsid of phages and the encoding DNA packaged in phage particles.^[^ ^96^ ^]^ The binding selection condition could be precisely controlled in vitro and in vivo. Phage display is a significant technological platform to engineer peptides or proteins because of its high‐throughput nature, ease to use, and rapidity.

A random phage peptide library is composed of a large number of individual phages displaying different peptides. DNA fragments encoding short peptides are synthesized first, which are then amplified or hybridized by PCR to form cloned fragments carrying appropriate restriction sites, and cloned into the region of phage capsid fusion proteins, to obtain a large number of recombinant phage‐displayed peptide libraries containing the expressed target genes.^[^ ^15^ ^]^ The key to the construction of a peptide library is the large storage capacity to include the possibility of almost all gene sequence combinations. This technology has shown great applications since its birth and has been broadly applied in diverse regions of nanomedicine science. The successful generation of high‐quality libraries depends on the success of phage display technology.

**Figure 7:**
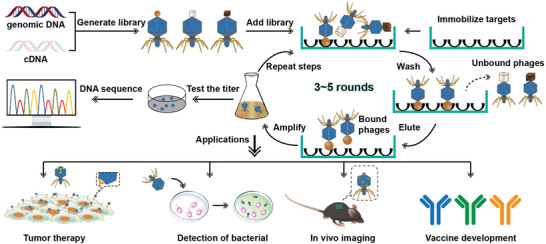
Use of a T7 phage based library to select targeting peptides for different biomedical applications.

The ordinary procedures to generate unique phages from T7 bacteriophage display libraries, also known as biopanning, are represented as follows.^[^ ^15^ ^]^ At first, a T7 phage library consisting of numerous bacteriophage clones is prepared, where every clone carries a single polypeptide or protein. Then the phage libraries are incubated with the targets.^[^ ^78^ ^]^ After incubation for a while, a washing buffer is then used for eliminating the unbound phages or phages with inferior binding force, leading to the retention of the phages with higher affinity. Then an elution buffer is used for collecting affinity phages.^[^ ^97^ ^]^ By infecting *E. coli*, the bound phages are amplified to create a pool of enrichment for the next rounds of biopharmaceutical libraries. As to selecting phages with high affinity, 3–5 rounds of biopanning would be performed strictly. Then, the enrichment pool is identified by enzyme linked immunosorbent assay (ELISA).^[^ ^48^, ^49^, ^98^ ^]^ Besides, biopanning methods can be divided into in vivo panning and in vitro panning, with targets being the tissues or organs in an animal body and biological materials in vitro, respectively. There are various practical applications in biomedical studies for the T7 phage display system, including understanding disease mechanisms, improving cancer detection, and developing drugs and vaccines (**Figure** **7**).

### 3.3 Generation of Antibody‐Displaying Libraries

The phage display technique was first introduced into the construction of an antibody library in 1989. The generation process of the antibody display library is to combine the amplification or synthesis of variable domain genes on heavy and light chains with the fragments of antibody cloning into phages.^[^ ^90^ ^]^ The detailed procedures are shown as follows. First, the biochemical method is used to extract the immune globulin mRNA and reverse transcription into cDNA. Suitable primers are chosen to use PCR technology for the DNA fragments amplification of light and heavy chains. Two kinds of chains are restructured randomly, and then imported into a specific carrier after enzyme digestion. Then the resulting phage is transfected into host cells. After that, the antibody gene is integrated into the phage coat protein gene with the helper phage, and the antibody is then expressed in the phage capsid as a fusion protein, to realize the combination with special immobilized antigens.^[^ ^99^ ^]^

The antibody‐displaying library is composed of lots of antibody fragment variants with a vast diversity of DNA sequences from natural repertoires^[^ ^90^ ^]^ or synthetic repertoires,^[^ ^100^ ^]^ forming full antibodies later. The library consists of different antibody fragments, including fragments of antigen binding (Fab) and single‐chain fragment variable (scFv) antibodies, providing considerable possibilities to identify specific binding molecules with high affinity, which have various functional performances.^[^ ^90^ ^]^

The Fab antibody library is constructed by displaying one strand of Fab (Fd) on the phage surface and another strand (light strand) into the vector. The two are then transfected into *E. coli*. One strand secreted into the periplasm matches another strand on the surface of phages to form a full Fab fragment fused with the capsid protein.^[^ ^90^ ^]^ For scFV library construction, first V_H_ and V_L_ genes are amplified respectively and then assembled into scFV by enzymatic digestion or ligation. Next, scFV is cloned inside the vector, which is transferred with target genes to *E. coli* for expression.^[^ ^90^ ^]^

## 4 Theranostic Use of T7 Phage

Bacteriophage display technology and biopanning method have been well reported in some reviews and books.^[^ ^69^, ^70^, ^101^, ^102^, ^103^, ^104^, ^105^ ^]^ Phage libraries could be applied for selecting polypeptides that bind to fixed proteins,^[^ ^106^, ^107^, ^108^, ^109^ ^]^ carbohydrates, nanoparticles,^[^ ^110^ ^]^ cells,^[^ ^111^ ^]^ and tissue.^[^ ^112^, ^113^, ^114^ ^]^ In vitro biopanning methods using T7 bacteriophages to obtain specific affinity polypeptides,^[^ ^41^, ^42^ ^]^ proteins^[^ ^40^ ^]^ or antibodies^[^ ^115^ ^]^ have several advantages such as high efficiency, low cost, and simple operation.^[^ ^116^ ^]^ In vivo biopanning techniques are proved significant to identify cell‐ or tissue‐affinity addresses.^[^ ^38^, ^117^ ^]^ After injection of phage libraries intravenously into mice, T7 phages are circulated, and some phages binding to specific organs or tissues are collected. The advantage of this technique is that it can identify regions with which the identified molecules interact and bind without an understanding on the mechanism of mutual contact in advance.

### 4.1 Detection of Serological Biomarkers

As we all know, traditional diagnostic technology of serological biomarkers needs many serum samples with low efficiency and high cost. Autoantibodies for different kinds of diseases have been observed in the serum of most patients, so biomarkers in the serum can be used to screen, diagnose, or monitor diseases.^[^ ^118^, ^119^ ^]^ By introducing the T7 phage display system into the detection of serological biomarkers, this new type of diagnostics can be more accurate, convenient, and efficient.^[^ ^23^, ^24^, ^25^, ^26^, ^27^, ^28^, ^29^, ^30^, ^31^, ^32^, ^33^ ^]^

For example, Talwar et al. isolated mRNA from leukocytes and bronchoalveolar lavage (BAL) cells of patients with nodule disease, and then extracted cDNA library of T7 phages.^[^ ^23^ ^]^ They constructed a microarray platform, and screened serum from lung cancer patients, cystic fibrosis subjects, and healthy controls.^[^ ^23^ ^]^ Next, 1000 Bayes models were built on the training sets from the microarray platform and the top 20 frequently significant clones were picked up. The results indicated that the cystic fibrosis specific clones were significantly correlated with the clinical value, such as body mass index (BMI), Forced Expiratory Volume 1% (FEV1%), and sweat chlorine test, at a high Pearson correlation. They demonstrated that immunological biopanning using the T7 phage display library could accurately identify cystic fibrosis‐specific serological markers for the first time, which turned out to have practical values for the development of molecular therapy.^[^ ^120^, ^121^ ^]^

Another example is a study by Li et al.^[^ ^28^ ^]^ To improve the efficiency of early diagnosis for lung tumor, they constructed a lung tumor protein chip based on biomarkers selected from the constructed lung cancer T7‐phage cDNA library. First, the antigen‐antibody reaction selected T7 phages with affinity against the serum antibodies from a human with and without lung tumors. After four rounds of biopanning, they obtained 2880 bacteriophages corresponding to lung cancer diseases. Twelve bacteriophages only bound to serum antibodies of lung cancer patients were screened through high‐throughput ELISA analysis. Then six kinds of precise or similar gene sequences were found by comparing them with known sequence genes in GeneBank. Finally, the human lung cancer protein chip was developed for quick and convenient diagnosis.^[^ ^28^ ^]^

**Figure 8:**
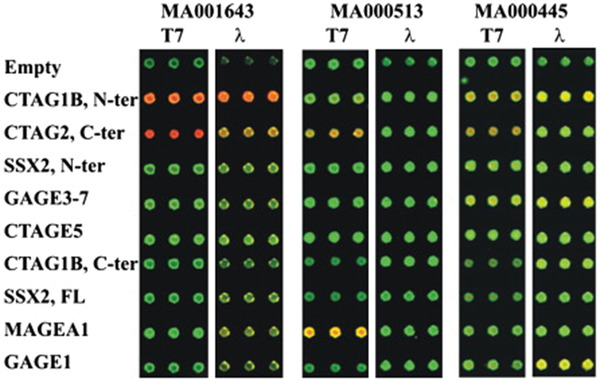
Antigen microarrays with T7 and *λ* phages displayed. Nine recombinant T7 and lambda phage clones related to tumor‐associated antigens expressing were amplified, and the serum of patients and monoclonal antibody were detected by Cy5‐ and Cy3‐labeled secondary antibody, respectively. The T7 phage vector was proved highly sensitive with antibodies of CTAG, MAGEA, and GAGE family members. Reproduced with permission.^[^ ^29^ ^]^ Copyright 2008, Elsevier.

Kalniņa et al. reported that a series of antigens were identified by rounds of T7 phages biopanning with serum from melanoma patients.^[^ ^29^ ^]^ This study tried to identify antigen‐induced B cell responses of the T7 and lambda vectors as well as to use autoantibody monitoring data to prepare phage display antigen microarrays. Fifteen tumor‐associated antigen family members were cloned into the phage display vector, and then they screened the serum of 22 melanoma patients with phages for tumor‐associated antigens. T7 vectors were proved highly sensitive to the antibodies of CTAG, MAGEA, and GAGE family members, as shown in **Figure** **8**. Besides, Luo et al. constructed the T7 phages’ genomic DNA library of mycobacterium tuberculosis, and screened the specific antigen binding to the positive serum of mycobacterium tuberculosis from the whole genome of mycobacterium tuberculosis to achieve higher specificity and sensitivity of serological diagnosis of tuberculosis.^[^ ^122^ ^]^

The combined use of multiple biomarkers has the potent capacity to promote the early diagnosis of diseases efficiently and accurately.^[^ ^28^ ^]^ Traditional diagnostic methods based on a single biomarker are proved imperfect diagnostic tools in clinical application due to their inefficiency, high cost, and sophisticated use.^[^ ^123^, ^124^ ^]^ Thus, multiple biomarkers and new test methods are expected to be developed.^[^ ^125^ ^]^ Protein chips depending on the T7 bacteriophage display system have a great potential in achieving high efficiency and miniaturization.

### 4.2 Detection of Bacterial Pathogens

**Figure 9:**
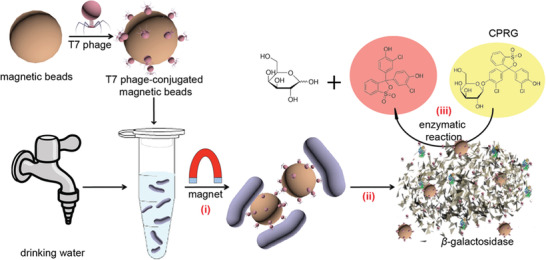
Schematic diagram of *E. coli* detection in contaminated water by applying magnetic separation technology based on T7 vectors. Three steps involved: i) Separating *E. coli* BL21 from contaminated water by utilizing modified magnetic beads; ii) Releasing *β*‐gal after the T7 phage infects *E. coli*; iii) Producing CPRG hydrolysis catalyzed by *β*‐gal. Reproduced with permission.^[^ ^31^ ^]^ Copyright 2015, American Chemical Society.

Detecting bacteria that caused foodborne illness quickly is an increasing need for public health.^[^ ^126^, ^127^ ^]^ T7 phage is an active biometric element, which is very suitable for quick bacterial detection with high sensitivity since there exist many advantages, including their specificity against host cells, low cost, high efficiency, and easy to modify foreign genes.^[^ ^128^ ^]^ Among all kinds of foodborne illnesses, drinking water pollution caused by pathogenic bacteria resulted in significant food safety and public health issues around the world.^[^ ^129^, ^130^, ^131^, ^132^ ^]^ *E. coli* has caused a high death rate and a high morbidity rate worldwide. To solve this problem, many efforts have been made.^[^ ^30^, ^31^, ^32^, ^33^, ^34^, ^35^, ^36^ ^]^ Chen et al. conjugated the T7 bacteriophage, a lytic phage with specificity against *E. coli*, with magnetic beads to capture and separate *E. coli* BL21 from drinking water rapidly and specifically.^[^ ^31^ ^]^ First, *E. coli* was separated from contaminated drinking water by utilizing T7 phage‐conjugated magnetic beads; Then, *β*‐galactosidase (*β*‐gal) was released from the bounded bacteria through phage‐mediated lysis. At last, chlorophenol red‐*β*‐d‐galactopyranoside (CRPG) was used to detect the presence of *β*‐gal. During these studies, they have detected *E. coli* with a concentration of 10^4^ CFU mL^−1^ in 2.5 h while there existed competing bacteria. **Figure** **9** represents the scheme of *E. coli* detection in contaminated water by applying the T7 phage‐based magnetic separation technology.

**Figure 10:**
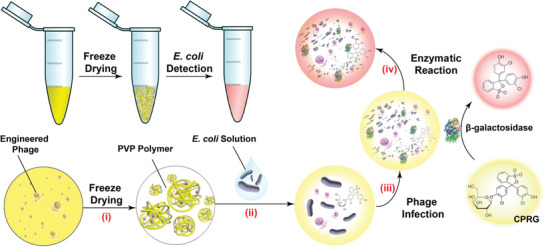
Scheme of *E. coli* detection by lyophilized engineering phages. There were four steps: i) detection kit freeze‐dried in a centrifuge tube, ii) lyophilized polymers dissolved by *E. coli* solution, iii) excessive expression of *β*‐gal caused by engineered phage infection, and iv) enzyme hydrolysis of CRPG to produce signals. Reproduced with permission.^[^ ^34^ ^]^ Copyright 2017, American Chemical Society.

Another example of *E. coli* detected by *β*‐gal in drinking water was also reported by Chen et al. Based on their previous research, the team used lyophilized engineered T7 bacteriophages to conduct the colorimetric tests of *E. coli* in water‐soluble polymers.^[^ ^34^ ^]^ The basic principle of this study is similar to their previous work. At the same time, this kind of commercial bacteria detection kit has advantages of more convenient use and transportation, lower cost, and higher efficiency, which makes it better for industrial production and use.^[^ ^34^ ^]^ **Figure** **10** shows the procedures to detect *E. coli* by lyophilized engineering phages.

**Figure 11:**
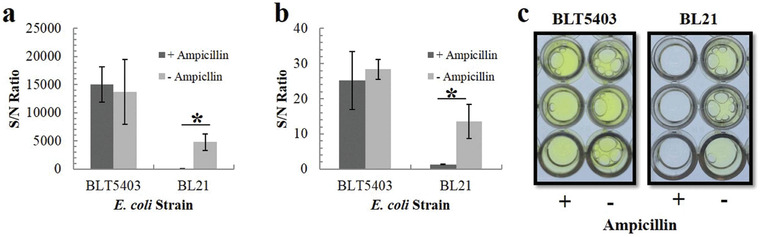
Antibiotic resistance detections and the signal/noise ratio of phoA activity. There were two *E. coli* strains in the presence or absence of ampicillin. a) PhosphaLight and b) pNPP. c) Photographs of plates containing pNPP substrate. Reproduced with permission.^[^ ^36^ ^]^ Copyright 2015, Royal Society of Chemistry.

Alcaine et al. reported a special T7 bacteriophage genetically modified by alkaline phosphatase (phoA) gene, which led to the excessive expression of phoA after T7 phage infected *E. coli*.^[^ ^36^ ^]^ If an *E. coli* isolate was resistant to ampicillin, a phage‐based probe could rapidly infect the *E. coli* and be amplified to produce a high signal to detect low levels of bacteria. Otherwise, the level of bacteria would be much lower and the phage probe could not produce the visible signal. By using this probe, they could find out whether the *E. coli* isolates were resistant to ampicillin using chemiluminescent or colorimetric substrates within 4.5 or 6 h, respectively.^[^ ^36^ ^]^ The detection results of antibiotic resistance are shown in **Figure** **11**. The level of *E. coli* strain BLT5403 detected by the phage‐based probe showed no significant difference with and without ampicillin, meaning *E. coli* strain BLT5403 had the antibiotic resistance. However, the level of *E. coli* strain BL21 detected by the phage‐based probe was much higher without ampicillin, suggesting that *E. coli* strain BL21 did not have the antibiotic resistance. This kind of phage‐based scheme may not require much investment and can be converted to other phages‐bacterial pairs for further testing.

**Figure 12:**
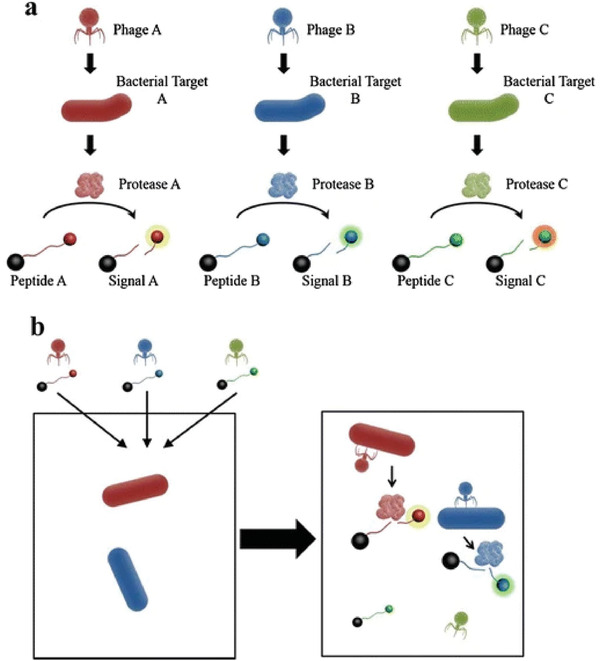
Schematic diagram of multiplex detection. a) Bacteriophages that target specific bacteria were biologically engineered to carry genes for specific proteases that identify specific peptides by corresponding fluorescence signals or specific peptide products. b) Different genetically engineered phages could be added to one sample at the same time to realize the simultaneous identification of different bacteria. Reproduced with permission.^[^ ^37^ ^]^ Copyright 2015, Springer Nature.

Except for sensing a single type of bacteria (*E. coli*), multiplex bacteria detection is also possible. Some studies have reported various detection schemes of *Salmonella* and *Listeria* by using phage components.^[^ ^133^, ^134^ ^]^ Alcaine et al. constructed a new T7 phage‐based strategy that used phage bioengineering to carry highly specific protease coded genes to detect its activity by specific peptides cleavage, thus achieving multiple detections of bacteria.^[^ ^37^ ^]^ First, genes of T7 phages were modified with the tobacco etch virus (TEV) protease gene. Second, after infection by T7 phage, TEV protease was expressed by *E. coli*. Finally, the TEV protease activity was utilized for *E. coli* detection in these samples. This principle of detection could also be applied to the mixture of different kinds of phage protease peptides, which enabled multiple bacterial detections and made it easy to use on various platforms. Indeed, bacteriophages that target specific bacteria were bioengineered to carry genes for specific proteases. After that, different genetically engineered phages could be added to one sample at the same time to realize the simultaneous identification of different bacteria. The process is shown in **Figure** **12**.

In summary, the bioengineering of T7 phages develops new strategies for specific and sensitive pathogens detection in multiple samples. Ongoing researches and new phage constructions will further expand the applications of phage‐based diagnostic technologies around the world.^[^ ^37^ ^]^

### 4.3 Targeted In Vivo Imaging

Phage display technology provides a unique platform to select new targeting peptides for imaging tumors as phages could be genetically modified with specific targeting peptide and simultaneously express multiple copies of these peptides.^[^ ^135^ ^]^ In the last few years, some peptides discovered by phage display have been optically labeled for the tumor imaging in vivo.^[^ ^136^, ^137^, ^138^ ^]^

**Figure 13:**
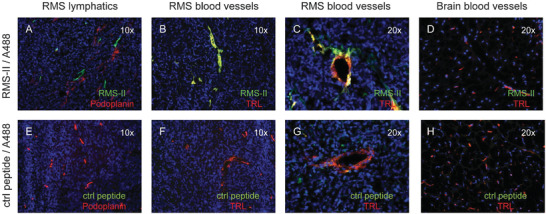
In vivo distribution of RMS‐II peptide. The tumor section was stained with avidin‐Alexa488 (A488), after the tumor‐bearing mice were injected with biotinylated RMS‐II peptide a–d) and unrelated biotinylated control peptide e–h), respectively. a) Staining of lymphatic vessels (red) and RMS‐II peptide (green). RMS lymphatics and RMS‐II peptide did not colocalize. b) Staining of tumor blood vessels (red) and RMS‐II peptide (green). The blood vessels of tumors colocalized with the RMS‐II peptide. c) Detail of staining of tumor blood vessels (red) and RMS‐II peptide (green). d) Staining of lectin (red) and RMS‐II (green). The brain blood vessels did not show accumulation of RMS‐II peptide. e–h) The control peptide injected into mice showed no accumulation in tumor and brain. Reproduced with permission.^[^ ^139^ ^]^ Copyright 2009, Wiley‐Liss, Inc.

Rhabdomyosarcoma (RMS) is a malignant tumor caused by abnormal cell growth, which is considered to be the most common soft tissue sarcoma in children. In 2009, Witt et al. reported two peptides selected from T7 phage‐displayed random peptide libraries could bind to RMS but not normal fibroblasts or skeletal muscle cells.^[^ ^139^ ^]^ One peptide named RMS‐I was an RGD‐containing peptide, and its binding to RMS was blocked by an antibody against *α*_v_ *β*_3_integrin. The other peptide named RMS‐II was a lymphatic binding peptide similar to Lymphatic Peptide 1 (LyP‐1), a previously reported peptide for tumor targeting.^[^ ^140^ ^]^ After the biotinylated RMS‐II peptide was injected into the RMS tumor‐bearing mice, a high fluorescence was detected in the tumor section, but little fluorescence was seen in the control tissue. **Figure** **13** shows the in vivo distribution of the RMS‐II peptide.

**Figure 14:**
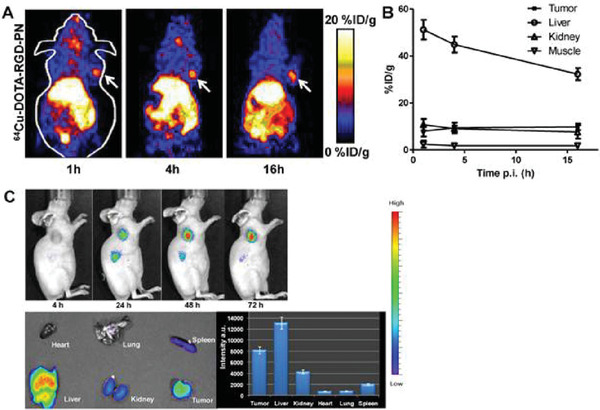
In vivo Imaging of U87MG Tumor‐Bearing Mice. A) PET images of nude mice bearing U87MG tumor at 1, 4, and 16 h postinjection of ^64^Cu‐DOTA‐Phage‐RGD. B) Time activity curve derived from multitime point PET study (*n* = 3). C) Optical images of U87MG tumor at 4, 24, 48, and 72 h after injection of Cy5.5‐Phage‐RGD. The major organs and tumor were picked up after 72 h. Reproduced with permission.^[^ ^141^ ^]^ Copyright 2011, Ivyspring International Publisher.

Besides, Li et al. reported a T7 phage nanoparticle with RGD peptides displayed on its surface targeting integrin *α*_v_ *β*_3_. The bifunctional chelator (BFC), 1,4,7,10‐tetraazadodecane‐*N*,*N*′,*N*″,*N‴*‐tetraacetic acid (DOTA) or 4‐((8‐amino‐3,6,10,13,16,19‐hexaazabicyclo [6.6.6] icosane‐1‐ylamino) methyl) benzoic acid (AmBaSar), was then conjugated onto the phage surface for ^64^Cu^2+^ chelation for positron emission tomography (PET) imaging.^[^ ^141^ ^]^ **Figure** **14** shows a whole body imaging after injection of the ^64^Cu‐radiolabeled phage. The integrin *α*_v_ *β*_3_ positive tumor U87MG was clearly visualized by ^64^Cu‐DOTA‐Phage‐RGD in PET images after 4 h circulation. There were little liver uptake and prominent tumor uptake after injection of Cy5.5‐Phage‐RGD. These results showed that PET based on phage particles can track the construction of organic nano‐platforms, which could be used for targeted tumor imaging.

### 4.4 Targeted Cancer Therapy

Traditional methods of cancer treatment have many disadvantages, including low precision, expensive costs, and reduced treatment efficacy.^[^ ^142^ ^]^ With the development of technology, significant advances have been made in theranostics, including therapeutic agents and multimodal imaging methods.^[^ ^143^, ^144^ ^]^ To produce nanoparticles with specific targeting ability, scientists always displayed affinity agents on the surface of particles.^[^ ^49^, ^144^ ^]^ Phage particles, a kind of virus particles, have a good prospect in targeted cancer therapy.^[^ ^145^ ^]^

The T7 phage is a mature protein‐expressing vector, which has drawn extensive attention for years and has made significant progress in targeted tumor treatment.^[^ ^146^, ^147^ ^]^ In the phage library, phages express different fusion proteins, and specifically targeted phages can be obtained by biopanning.^[^ ^52^, ^53^, ^135^, ^148^, ^149^ ^]^ The interaction mechanism between phages and cancer cells is considered energy‐dependent.^[^ ^150^ ^]^

**Figure 15:**
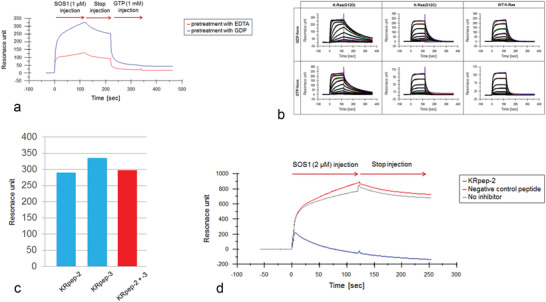
The binding activity of polypeptides toward K‐Ras measured by the SPR method. a) Binding activity of SOS1 pretreated with EDTA or GDP. b) Binding abilities of KRpep‐2. c) Competitive experiments of krpep‐2 and krpep‐3 overlapping binding sites on K‐Ras. There are three experimental groups: KRpep‐2, KRpep‐3, and KRpep‐2 + KRpep‐3. d) Binding competition measured between SOS1 and KRpep‐2. Negative control peptides, noninhibitor, and KRpep‐2 were incubated with SOS1 in cells. Reproduced with permission.^[^ ^42^ ^]^ Copyright 2017, Elsevier.

K‐Ras is a significant growth driver in different kinds of tumors so that it seems to be one of the most efficient drug targets. In 2017, Sakamoto et al. reported that K‐Ras(G12D) inhibitory peptides were screened through biopanning of T7 bacteriophage libraries.^[^ ^42^ ^]^ T7 phages with high affinity to K‐Ras were screened from a random T7 phage library, and the inhibition ability of affinity phages to K‐Ras was measured. Compared with wild‐type K‐Ras, the affinity peptides represented up to 10 times of inhibitory ability to K‐Ras. Among all of the peptides, the Krpep‐2d peptide inhibited the proliferation of cancer cells and the downstream signal of K‐Ras.^[^ ^41^ ^]^ **Figure** **15** shows the binding abilities of polypeptides to K‐Ras proteins measured by surface plasma‐resonance (SPR), which illustrates the overlapping binding sites of SOS1 and Krpep‐2 proteins on K‐Ras (G12D).^[^ ^42^ ^]^

**Figure 16:**
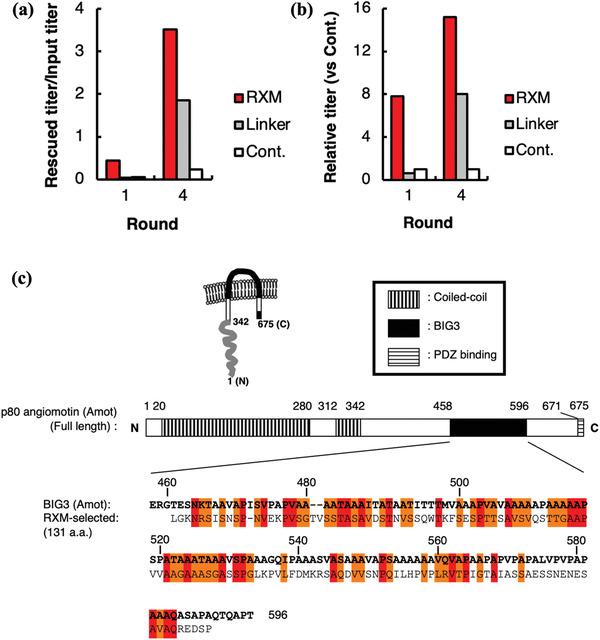
RXM recognition protein biopanned by T7 phage display technology based on a microplate. a) RXM affinity peptides selected by T7 phage library biopanning. Fixed biological RXM or biotin ligands were incubated with the T7 bacteriophages, respectively, at 25 °C for 3 h. Then unbinding T7 phages were washed off, and the T7 phages with high affinity were collected and incubated with the host, and then phage titer was obtained by counting plaque. The control group was the abiotic RXM. b) Relative phage titers compared with that of controls. c) Diagram of the Amot receptor. Similarities between RXM affinity peptides and BIG3 were shown. The identifications were highlighted in red and the similarities in orange. Reproduced with permission.^[^ ^51^ ^]^ Copyright 2015, Elsevier.

Roxithromycin (RXM) is a semisynthetic macrolide antibiotic with antiangiogenic activity in solid tumors, making it an efficient drug target. Takakusagi et al. found that RXM bound to the extracellular domain on angiomotin (Amot) as a common binding site with angiostatin (Anst), leading to inhibition of angiogenesis‐dependent tumor growth and metastasis.^[^ ^51^ ^]^ First, they selected the RXM affinity peptide by biopanning with the T7 phage library. Then three different biopanning strategies were used to identify RXM‐recognizing polypeptides independently. **Figure** **16** represents RXM recognition protein biopanned by T7 phage display technology based on the microplate.^[^ ^51^ ^]^ The bioinformatics analysis indicated that Amot could bind to RXM. Furthermore, the results of T7 phage biopanning showed that RXM was bound to the Amot extracellular region to realize inhibition of growth and metastasis of angiogenesis‐dependent tumors, which may help understand the antiangiogenesis mechanism of RXM on a molecular level.

**Figure 17:**
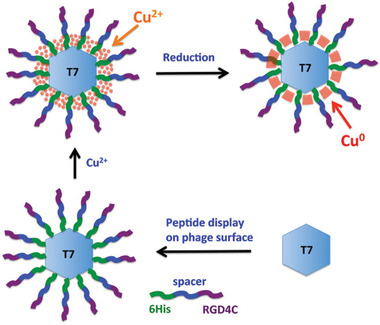
The schematic diagram for the construction of recombinant phage loaded with low‐concentration copper ions efficiently. First, 6His‐spacer‐RGD4C peptide was displayed on the phage surface; then, copper ions were loaded near the surface; finally, the Cu‐phage nanoparticle was formed by reducing copper ions to copper. Reproduced with permission.^[^ ^49^ ^]^ Copyright 2012, American Chemical Society.

T7 phage can be widely used in targeted cancer therapy by displaying affinity reagents on the surface, which are conjugated to cancer cells directly. For example, Dasa et al. reported that low‐concentration copper ions could be loaded in T7 phage to form the recombinant T7 bacteriophage without reducing the targeting specificity. They displayed hexahistidine (6His) peptide and RGD4C sequence on the T7 phage to form a single peptide (6His‐spacer‐RGD4C), which targeted cancer cells by а_V_ *β*_3_ integrin. The copper ions were then reduced to copper to form a copper‐loaded T7 phage with high stability, which prevented copper separated from T7 phage nanoparticles and maintained the T7 phage structure even under very harsh conditions. Through ligand‐mediated transmembrane transport, copper‐hybridized T7 phage particles would be selectively ingested by MCF‐7 cancer cells, and the intake rate was 1000 times higher than the control group. **Figure** **17** represents the schematic diagram for the construction of recombinant phage loaded low‐concentration copper ions efficiently.^[^ ^49^ ^]^

**Figure 18:**
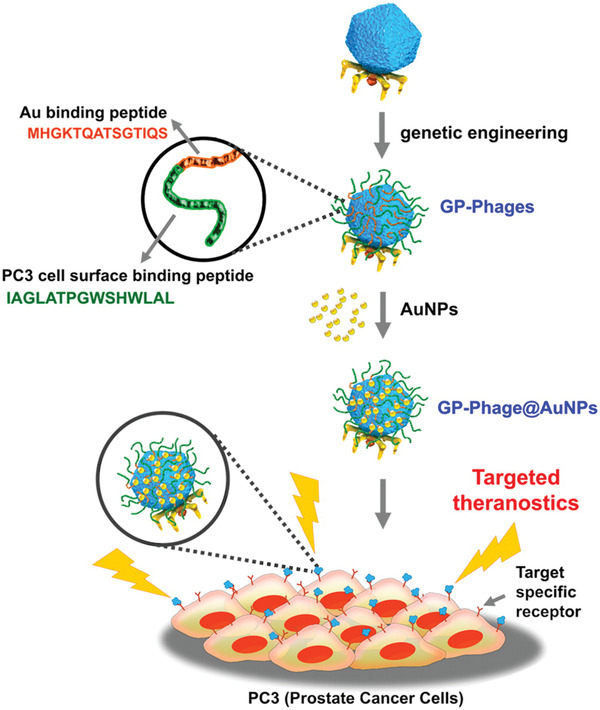
Principle of cancer‐affinity PTT therapy using T7 phage. T7 phage was displayed with gold binding and PC3 cell surface binding polypeptides on capsid by gene modification. AuNP nanocrystals were applied on the surface of T7 phage to achieve targeted photothermal therapy. Reproduced with permission.^[^ ^50^ ^]^ Copyright 2015, American Chemical Society.

Besides, phage‐based nanomedicine could also be employed for treating cancer by photodynamic therapy (PDT) or photothermal therapy (PTT).^[^ ^151^, ^152^, ^153^ ^]^ Some studies reported that T7 bacteriophages with the RGD motif displayed showed a high affinity to human transferrin.^[^ ^154^ ^]^ For example, Oh et al. constructed a recombinant T7 phage whose capsid protein surface was bound with both PC3 cell surface binding peptide and Au‐binding peptide.^[^ ^50^ ^]^ Then, the resultant phages, termed GP‐phages, were modified with Au nanoparticles (AuNPs) to form GP‐phage‐AuNPs. Under certain light intensity, the light‐to‐heat conversion ability of AuNPs made them efficient heat sources for hyperthermic damage of specific cancer cells.^[^ ^155^, ^156^, ^157^ ^]^ Some studies have discovered that particle‐free crosslinked AuNPs could significantly increase the efficiency of photothermal therapy.^[^ ^158^, ^159^, ^160^ ^]^ In the study by Oh et al., GP‐phage‐AuNPs killed prostate cancer cells rapidly under the low intensity of light irradiation, while both citrate‐stabilized AuNPs and nontargeted AuNP clusters caused the death of few cancer cells. **Figure** **18** illustrates the principle of phage‐based cancer‐affinity PTT therapy.

To conclude this section, the T7 phage system has extensive usage in the targeted cancer therapy due to the advantages such as ease in generating them, facile display of affinity peptides, and lack of toxicity. It can be potentially used in direct conjunction with photodynamic, photothermal, or gene therapy.

### 4.5 Targeted Antibacterial Therapy

The use of traditional antimicrobial agents in the body can cause serious problems, including dose‐dependent systemic toxicity and bacterial resistance, impeding the healing process.^[^ ^123^ ^]^ Actually, antibacterial drugs are one of the most misused drugs because of their low cost and easy accessibility.^[^ ^124^ ^]^ A study from Liu et al. indicated that the potential packaging between phage and it host showed an antibiotic resistance genes (ARG) preference rather than occurring randomly.^[^ ^161^ ^]^ They isolated and characterized two T7‐like lytic bacteriophages (HZP2 and HZ2R8) that could infect multidrug‐resistant *E. coli*. The morphology and genomic analysis indicated that both phage HZP2 and HZ2R8 were evolutionarily related and their genomes did not encode ARGs. However, ARG‐like raw reads were detected in offspring sequencing data with a different abundance level implying that potential ARG packaging had occurred. The study indicated that these structurally similar phages possessed similar characteristics and packaging during phage–host interaction that displayed an ARG preference rather than occurring randomly. Nowadays, many efforts are taken to realize targeted antibacterial therapies based on bacteriophages with the aim at solving the bacterial resistance and toxicology issues caused by traditional antimicrobial agents.^[^ ^125^, ^162^, ^163^, ^164^, ^165^ ^]^

**Figure 19:**
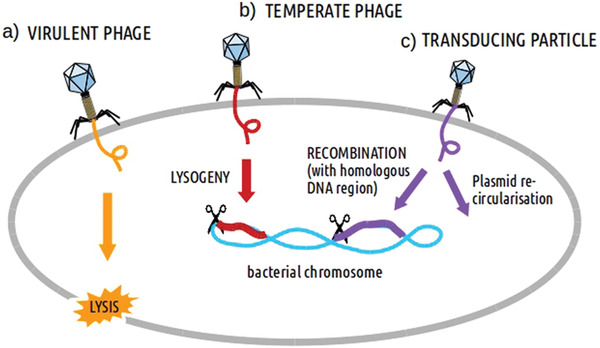
Three different kinds of processes for phages to infect bacteria and inject DNA into it. a) Virulent phages break open bacteria. b) Temperate phages cause lysogeny. c) Transducing phages achieve DNA recombination/transduction. Reproduced with permission.^[^ ^168^ ^]^ Copyright 2018, Springer Nature.

Most phages package their nucleic acid in capsid close to crystalline concentration to protect their genetic materials.^[^ ^166^ ^]^ This highly condensed genome is then injected into host bacteria through a process called “ejecting.”^[^ ^79^, ^81^ ^]^ Lots of bacteriophages deliver the genome by using a special compound (usually in the tail) without breaking their integrity. One study showed that T7 phages could identify lipopolysaccharides (LPS) in crude *E. coli* strains through fibers.^[^ ^167^ ^]^ As the central receptor of T7 phages, rough LPS drives the ejection of DNA in vitro. Interactions with the receptor cause the fibers to tilt and internal tail channels to open, allowing the release of DNA and possibly internal head proteins by unlocking the nozzle domain. **Figure** **19** shows three different kinds of processes for phages to infect bacteria and inject DNA into them.^[^ ^168^ ^]^

The maturity of genetic engineering technology has enabled phages to serve as excellent manufactured probes for targeted antibacterial therapy, which has been developed rapidly recently.^[^ ^43^, ^44^, ^45^ ^]^ T7 phage has been considered to be a potential method to reduce the impact of foodborne pathogens. Nitin et al. evaluated the antimicrobial activity of T7 phage, and the results showed an improved antimicrobial property against *E. coli* BL21.^[^ ^45^ ^]^

Besides, the studies on interactions between bacteriophages and bacteria in biofilms have developed a lot.^[^ ^169^, ^170^ ^]^ Phage has been used as an antibiofilm agent in various industrial and clinical diagnoses as well as phage therapy,^[^ ^170^ ^]^ the filtration membranes^[^ ^171^ ^]^ and biofilm‐infected medical devices,^[^ ^172^, ^173^, ^174^ ^]^ due to its lysis of bacteria.^[^ ^175^, ^176^, ^177^ ^]^ For example, Pei et al. constructed a recombinant T7 phage encoding an active lactonase enzyme to quench quorum sensing.^[^ ^43^ ^]^ Bacterial cells communicate with each other via acyl to serine lactones (AHLs), which seems to be significant for biofilm formation.^[^ ^178^, ^179^ ^]^ Their data demonstrated that aiiA lactase expressed by T7 phage could efficiently damage AHLs of various bacteria. It has then been proved that engineering T7 phage could inhibit the formation of biofilms if added it into the mixed biofilms containing *E. coli* and pseudomonas aeruginosa.^[^ ^43^ ^]^ This quorum quenching phage, which lyses host bacteria and expresses bacteriostatic enzymes, may be a new antiseptic and antibacterial agent because it can affect the biofilms of various bacterial communities in both industrial and clinical situations.

Since bacteriophages are active elements, strict quality control must be implemented to ensure no adverse effects. Although T7 phages cause much less bacterial lysis than antibiotics do, the lysis is enough for the targeted antibacterial therapy. The problem needed to be solved in the future is the possibility of using several bacteriophage mixtures to establish an ideal drug delivery pathway and to modify its genes to inactivate the bacteria‐resistant genes.^[^ ^180^, ^181^, ^182^, ^183^ ^]^

### 4.6 Targeted Gene Therapy

Phage vectors have many promising characters compared with viral and nonviral DNA delivery systems. They are nanoscale natural systems that carry foreign DNA insertion and efficient packaging.^[^ ^184^ ^]^ It is cost‐effective and straightforward to produce and purify phage particles on a large scale.^[^ ^185^ ^]^ Several phages promote DNA transfer through the protein transduction domain of immunodeficiency virus type 1 (Tat peptide).

**Figure 20:**
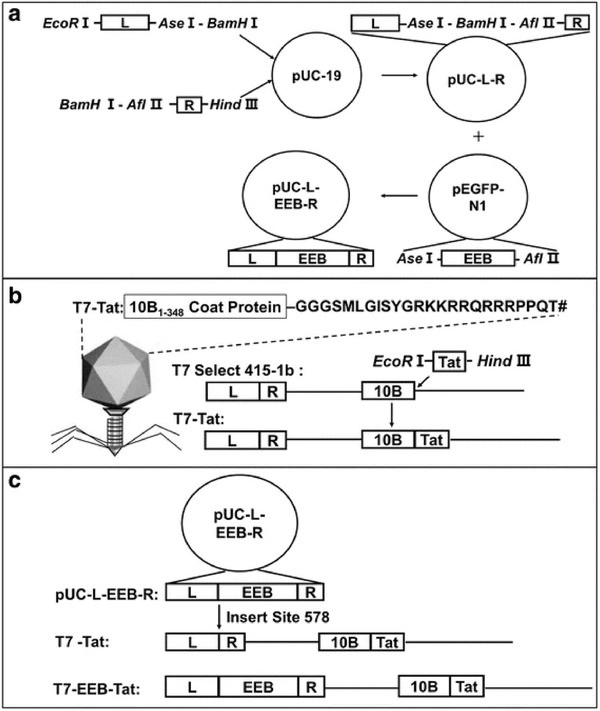
Construction of engineered T7 plasmid. a) Construction of plasmid pUC‐L‐EEB‐R. Plasmid pUC‐L‐R was constructed by cloning an amplified homologous arm into pUC‐19. Furthermore, pUC‐L‐EEB‐R was constructed by cloning complete EEB into pUC‐L‐R. b) Construction of recombinant T7‐Tat. The engineered T7‐Tat phage was artificially synthesized by inserting the selected 415‐1b genome of T7. c) Construction of recombinant T7‐EEB‐Tat with the surface displaying a Tat peptide. Reproduced under the terms of a Creative Commons Attribution 4.0 International License.^[^ ^21^ ^]^ Copyright 2018, The Authors. Published by Springer Nature.

T7 bacteriophage can tolerate the insertion of foreign gene sequences of a certain length into its genome. Moreover, it has been reported to deliver DNA into cells as a competent vector. However, the lack of eukaryotic targeted receptors leads to the low efficiency of gene transfer, limiting the use of T7 phage in delivering DNA vaccines. Previous studies have shown that surface Tat targeting peptides can improve the transfer efficiency of phage‐mediated DNA. For instance, Xu et al. used T7 phage as a DNA delivering vector to promote cell internalization.^[^ ^21^ ^]^ They constructed a recombinant T7 phage, named T7‐EEB‐Tat, with Tat peptide modified and eukaryotic expression box (EEB) carried. The recombinant T7‐EEB‐Tat phage promoted phage‐targeted expression of reporter genes in eukaryotic cells. **Figure** **20** shows the construction of the engineered T7 plasmid. Vectors are crucial to the success of gene delivery.^[^ ^186^, ^187^ ^]^ Compared to the wide use of lambda phages in gene therapy,^[^ ^184^, ^185^, ^186^, ^187^, ^188^, ^189^, ^190^, ^191^, ^192^, ^193^, ^194^, ^195^, ^196^, ^197^, ^198^, ^199^, ^200^, ^201^, ^202^, ^203^ ^]^ the application of T7 phage in vaccine design is considered the potential for promoting targeted gene delivery in gene therapy due to its ability of surface display.

### 4.7 Other Targeted Therapies

Except for the most widely used targeted cancer and antibacterial therapies, the therapies of various diseases have also been reported using T7 phage. Discovering polypeptides or proteins targeting diseases could be achieved through the T7 phage display system. Many studies have showed us the significant advantages of T7 phage display in vaccines^[^ ^21^, ^46^, ^47^ ^]^ and targeted therapy for various diseases.^[^ ^19^, ^188^ ^]^

**Figure 21:**
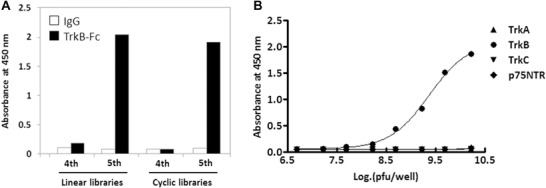
Biopanning results of random T7 bacteriophage display libraries. a) Binding specificity of T7 phages to TrkB‐Fc and IgG measured by ELISA. There existed two libraries, linear libraries and cyclic libraries. b) Binding activities of monoclonal phage BM17. The T7 phage binding affinities to p75NTR‐Fc, TrkA‐Fc, TrkB‐Fc, and TrkC‐Fc were measured. Reproduced with permission.^[^ ^53^ ^]^ Copyright 2017, Elsevier.

A novel tropomyosin receptor kinase B (TrkB) agonist was identified using T7 phage biopanning, reported by Ohnishi et al.^[^ ^53^ ^]^ TrkB is a well‐known brain‐derived neurotrophic factor (BDNF) receptor, which shows the ability to regulate the survival, maturation, and development of neurons, making it a good target for the central nervous system diseases treatment. The sequence of BM17d99 peptide generated by Ohnishi et al. was not similar to that of BDNF, but with a high affinity to TrkB. Then a dimeric Fc‐fused TrkB (TrkB‐Fc) was synthesized to induce the homodimerization and activation of TrkB. HEK293 cells expressing Trk‐B were treated with the dimeric TrkB‐Fc, leading to the phosphorylation of TrkB. This result suggested that the dimeric peptides have helped promote the homodimerization of TrkB. **Figure** **21** illustrates the biopanning results of random T7 bacteriophage display libraries against TrkB‐Fc.

Except for the central nervous system diseases, the T7 phage display system has also been applied to inflammatory diseases. Cytidine triphosphate synthetase 1 (CTPS1), an enzyme produced in activated lymphocytes, could promote the catalytic transformation from uridine triphosphate (UTP) to cytidine triphosphate (CTP). Since CTP is one of the direct precursors of RNA biosynthesis and is involved in the synthesis of polysaccharides, size expansion and proliferation of lymphocytes in inflammatory diseases can be inhibited by the CTPS1 inhibition. Recently, Sakamoto et al. reported the peptide CTpep‐3 binding to CTPS1 screened by random T7 phage libraries.^[^ ^42^ ^]^ Through surface plasmon resonance (SPR) analysis, CTPS1 was confirmed to bind to CTpep‐3 selectively. Through enzyme analysis, CTPS1 was found to be selectively inhibited by CTpep‐3. What's more, the results of the enzyme inhibition experiment and hydrogen deuterium exchange mass spectrometry (HDX‐MS) showed that CTpep‐3 could bind to the amidoligase (ALase) domain on CTPS1.

Besides, foot–mouth disease or aftosa disease is febrile and dangerous, causing substantial economic losses globally. Xu et al. reported recombinant T7 phage nanoparticles displaying G‐H (the primary neutralizing antigenic site) peptide (T7‐GH) to be a promising vaccine.^[^ ^48^ ^]^ First, the G‐H gene was inserted into the T7 vector to construct the recombinant phage T7‐GH. Then, Dot‐ELISA, Western blot, and SDS‐PAGE methods were used to detect the characteristic of T7‐GH phage nanoparticles. Then they found that antigen‐specific immune responses were induced by recombinant nanoparticles in pigs. Pigs immunized with T7‐GH produced antibodies and showed similar antigen‐specific lymphocyte proliferation as the PepVac vaccine group but less than the InactVac vaccine group. Moreover, the pigs immunized with T7‐GH produced neutralizing antibody responses and showed weaker responses than the InactVac group but stronger than PepVac group. InactVac and PepVac were both commercially available FMDV vaccines.

**Figure 22:**
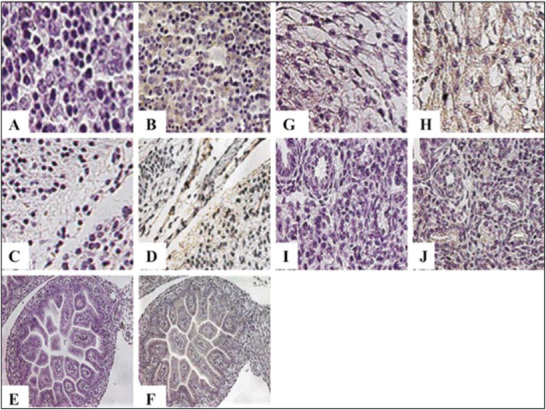
Immunostaining imaging of the fetal tissues based on the T7 phage . After tail vein injection of a T7 phage‐displayed C‐X7‐C peptide library, fetuses were harvested and immobilized in a 10% formalin solution. An antibody against T7 phage was used as the primary antibody for the staining followed by a secondary horseradish peroxidase (HRP)‐conjugated polyclonal antibody. Parts (B), (D), (F), (H), and (J) show the stained fetal liver, brain, intestine, heart, and lung after the T7 phage injection, respectively. Parts (A), (C), (E), (G), and (I) showed the related controls and the injections with phosphate buffer saline. Reproduced with permission.^[^ ^38^ ^]^ Copyright 2004, Future Science.

Most reported results are about the adult tissue targeting by applying the T7 bacteriophage display system; however, there exist fewer reports on using a phage display system to locate fetal tissues. Targeting fetal tissue in utero can significantly help correct many genetic and metabolic diseases, which makes it meaningful to apply T7 phage display technology by means of the maternal systemic circulation. After systemic circulation, the distribution of the library in fetal tissues in pregnant mice was reported by Srivastava et al.^[^ ^38^ ^]^ Their data suggested that T7 bacteriophages could penetrate the placental obstacles and then possibly arrive at the fetal tissue. Thus, the T7 phage display library was proved useful to design targeted therapy of uterine tissue for locating fetal tissue through maternal blood circulation.^[^ ^38^ ^]^ **Figure** **22** represents the immunohistochemical staining of fetal tissues after T7 phage is injected through the intravenous tail vein.

In summary, the T7 phages play a significant role as theranostic nanoparticles in precision medicine. Furthermore, the central idea of treatment applications includes first finding the targets of the disease and then obtaining the target‐binding affinity peptides through biopanning. Finally, the peptides, alone or displayed on phages or other nanoparticles, serve as the targeting inhibitors and block the signal transmission pathways.

## 5 Perspectives and Challenges

The full applications of phage display technology in nanomedicine are becoming a reality. T7 phage systems have drawn colossal attention due to their attractive properties, including high efficiency and exceptional stability. Through genetic engineering, specific peptides can be displayed on phages after the affinity peptides or affinity proteins are obtained through biopanning against the phage‐displayed libraries. The T7 phages can enter cells without showing toxicity, laying the foundation for using them for precision medicine. One of the reasons for the current treatment techniques to be ineffective lies in the lack of target specificity for the medicines, which can be addressed by phage display technology.

Although phages have advanced nanomedicine, there are still some problems that limit their further development and applications. For example, there exists a big gap between humans’ treatments and animal trials. What is more, although phages have been widely used as nano drugs in animal experiments, little research has been done on phage internalization into cells in human bodies. As a result, scientists knew a little about phage immune responses and cellular interactions in humans. Therefore, more corresponding studies are required to investigate the interaction between phages and the body as well as the stability of phages in different environments.

It is also very promising to assemble phages into 3D scaffolds to achieve tissue regeneration. This has been achieved in filamentous phages such as M13 through the development of layer‐by‐layer assembly or dip‐pulling methods.^[^ ^189^, ^190^, ^191^, ^192^ ^]^ However, a scaffold based on T7 phage nanoparticles has not been well investigated. Since a scaffold made of T7 nanoparticles can be potentially used to direct stem cell differentiation and regenerate tissue, efforts should be made to find ways of assembling them into 3D matrix or scaffolds.

Phage can be integrated with CRISPR/Cas9 assisted genome engineering technique to widen its applications in biomedicine. Success has been made in T4 phage system.^[^ ^193^, ^194^ ^]^ For instance, Duong et al. has achieved >99% editing rates of multiple functional phages for further applications in the fields of disease therapy, biocontrol, and precise diagnostics, such as treating infectious diseases caused by multidrug resistant antibiotic bacteria and detecting foodborne pathogens.^[^ ^194^ ^]^ The previous results of genome editing of phage T4 might be extended to other phages for phage therapy applications in general.^[^ ^193^ ^]^ However, little has been done in T7 phage system. Because T7 is similar to T4, we believe that CRISPR/Cas9 assisted genome engineering technique can be applied to T7 phage system by using two adjacent spacers for genome editing.

The recent introduction of phages into human bodies for disease therapy has shown that they do not elicit undesired side effects.^[^ ^195^, ^196^, ^197^, ^198^ ^]^ For example, Grygorcewicz et al. reported a patient with cystic fibrosis treated with three‐phage cocktail therapy, which showed that phages could reduce biofilm biomass in a human urine model for treatment of urinary tract infection (UTI).^[^ ^198^ ^]^ Moreover, now phages are known to exist in our human body to maintain our normal health.^[^ ^199^ ^]^ Therefore, it will be exciting to study the use of T7 phage in the diagnosis and treatment of challenging human diseases such as cancer in clinics. Extensive effort should be made to understand the fate of T7 phage in humans after being introduced into the body by different routes as well as the engineering of T7 phage into both a drug for targeted therapy and a probe for targeted imaging.

In general, the phage display technology has a reliable application in targeted theranostics of tumor cells, targeted detection of pathogens, targeted antibacterial therapy, and detection of bioactive substances, among others. These challenges, once overcome, will push the limit of the applications of the T7 phage in precision medicine. So far, no human preclinical/clinical study of T7 phages has been reported.^[^ ^200^, ^201^ ^]^ The large‐scale manufacture of the T7 phage could be done by infecting its host bacteria in a fermenter. The phage community was encouraged by the exciting news that other phage preparations (e.g., phage cocktails) have been found successful in treating diseases in human body such as fighting superbugs.^[^ ^202^, ^203^, ^204^, ^205^, ^206^, ^207^, ^208^, ^209^, ^210^, ^211^ ^]^ More efforts should be made to explore the use of T7 phage, engineered with improved functions, in theranostics. Once cases of phage therapy using engineered T7 phage are demonstrated in clinical trials, such T7 phages might have potential of being clinically approved by US Food and Drug Administration (FDA).

## 6 Summary

The current review has thoroughly discussed the multiple benefits of using the T7 phage as a biomaterial in nanomedicine, including the discovery of disease targeting peptides, detection of bacterial pathogens, quantification of serological biomarkers, targeted antibacterial therapy, and cancer therapy, among many others. Compared with the commonly used M13 phage system, T7 phage has excellent stability, grows fast, and can display larger peptides or proteins, making it a better vector or nano‐biomaterial for disease diagnosis and treatment. However, there still exist several problems to be solved, from understanding the immune response, assembly into higher‐ordered structures, integrating with gene editing systems, to pinpointing the fates in human bodies. More studies on the T7 phage as a targeting theranostic bio‐nanoparticle have to be made to achieve progress in precision medicine.

## Conflict of Interest

The authors declare no conflict of interest.
